# Structure and Stability of the Dimeric Triosephosphate Isomerase from the Thermophilic Archaeon *Thermoplasma acidophilum*

**DOI:** 10.1371/journal.pone.0145331

**Published:** 2015-12-28

**Authors:** Sang Ho Park, Hyoun Sook Kim, Mi Seul Park, Sojin Moon, Mi Kyung Song, Han Su Park, Hyunggu Hahn, Soon-Jong Kim, Euiyoung Bae, Hyun-Jung Kim, Byung Woo Han

**Affiliations:** 1 Research Institute of Pharmaceutical Sciences, College of Pharmacy, Seoul National University, Seoul, Korea; 2 Department of Agricultural Biotechnology, Seoul National University, Seoul, Korea; 3 Department of Chemistry, Mokpo National University, Chonnam, Korea; 4 College of Pharmacy, Chung-Ang University, Seoul, Korea; Yale University School of Medicine, UNITED STATES

## Abstract

*Thermoplasma acidophilum* is a thermophilic archaeon that uses both non-phosphorylative Entner-Doudoroff (ED) pathway and Embden-Meyerhof-Parnas (EMP) pathway for glucose degradation. While triosephosphate isomerase (TPI), a well-known glycolytic enzyme, is not involved in the ED pathway in *T*. *acidophilum*, it has been considered to play an important role in the EMP pathway. Here, we report crystal structures of apo- and glycerol-3-phosphate-bound TPI from *T*. *acidophilum* (TaTPI). TaTPI adopts the canonical TIM-barrel fold with eight α-helices and parallel eight β-strands. Although TaTPI shares ~30% sequence identity to other TPIs from thermophilic species that adopt tetrameric conformation for enzymatic activity in their harsh physiological environments, TaTPI exists as a dimer in solution. We confirmed the dimeric conformation of TaTPI by analytical ultracentrifugation and size-exclusion chromatography. Helix 5 as well as helix 4 of thermostable tetrameric TPIs have been known to play crucial roles in oligomerization, forming a hydrophobic interface. However, TaTPI contains unique charged-amino acid residues in the helix 5 and adopts dimer conformation. TaTPI exhibits the apparent T_d_ value of 74.6°C and maintains its overall structure with some changes in the secondary structure contents at extremely acidic conditions (pH 1–2). Based on our structural and biophysical analyses of TaTPI, more compact structure of the protomer with reduced length of loops and certain patches on the surface could account for the robust nature of *Thermoplasma acidophilum* TPI.

## Introduction

Triosephosphate isomerase (TPI or TIM) is a functionally and structurally well-known enzyme that plays a crucial role in glycolytic and gluconeogenic metabolism. TPI accurately and efficiently interconverts dihydroxyacetone phosphate (DHAP) and glyceraldehyde-3-phosphate (GAP). Missense mutations of TPI genes result in TPI deficiency through loss-of-function [1]. TPI deficiency has been known to cause metabolic diseases, glycolytic enzymopathies, in which neurological pathogenesis is uniquely severe [2]. Pathogenic TPI deficiency mutations dramatically affect TPI activity owing to either catalytic interruption or oligomeric state alteration [3–5].

*Thermoplasma acidophilum* is one of the most acidophilic organisms among known thermophilic archaea. It optimally grows at 55–60°C and pH 0.5–2. For glucose degradation, *T*. *acidophilum* has been known to utilize not only non-phosphorylative Entner-Doudoroff (ED) pathway but also canonical glycolysis/gluconeogenesis pathway (Embden-Meyerhof-Parnas pathway) [6]. Although one of the glycolysis/gluconeogenesis pathway enzymes, phosphofructokinase, has not been identified in *T*. *acidophilum*, TPI has been considered to play a crucial role in the glycolysis/gluconeogenesis pathway in *T*. *acidophilum*.

TPI is a representative α/β protein with eight α-helices and eight β-strands connected by loops, forming a TIM-barrel fold [7]. The active site of TPI is highly conserved in all kingdoms and located inside of the TIM-barrel fold with three catalytic residues (Lys9, His89, and Glu137 in TaTPI numbering). Most TPIs are optimally active in their dimeric forms. In contrast, archaeal TPIs that withstand high temperatures are tetrameric in their active states [8]. Thermostable proteins have been known to adapt to high temperature in various ways: increased electrostatic interactions, hydrogen bonds, and hydrophobic effects, resulting in fortified subunit contacts, more compact packing, higher oligomerization state, and two-state equilibrium reversibility [9–13].

In the Protein Data Bank (PDB), structures of one bacterial TPI (*Thermotoga maritima;* Tm) and three archaeal TPIs (*Pyrococcus woesei;* Pw, *Thermoproteus tenax;* Tt, and *Methanocaldococcus jannaschii;* Mj) that have been deposited are in tetrameric form. *P*. *woesei*, *M*. *jannaschii*, and *T*. *acidophilum* belong to Euryarchaeota and *T*. *tenax* is affiliated to Crenarchaeota in phylogeny. Among tetrameric TPIs in PDB, tetrameric conformation of bacterial TmTPI is maintained by disulfide bonds between two classical TPI dimers [8]. In archaeal TPIs, helix 4 and helix 5 in the tetrameric interface are of key importance for their tetrameric forms. Especially, helix 5 in thermostable TPIs has been considered as a major contributor for tetramer formation via dominant hydrophobic effects [14].

In this work, we report crystal structures of apo- and glycerol-3-phosphate (G3P)-bound TaTPI, each representing open and closed form. Unlike other thermostable archaeal TPIs, TaTPI forms a stable dimer in solution, which we confirmed using analytical ultracentrifugation and size-exclusion chromatography. We also show the effect of pH on the secondary structure and temperature-induced unfolding of TaTPI. Through systematic comparison of TaTPI with available dimeric and tetrameric TPIs, we suggest that TPI stabilization patches can be targeted for the design of more stable TIM-barrel fold proteins.

## Materials and Methods

### Cloning, expression, and purification of TaTPI and MjTPI

Full-length sequence of *Thermoplasma acidophilum* TPI (TaTPI, 216 amino acid residues) and *Methanocaldococcus jannaschii* TPI (MjTPI, 219 amino acid residues) were amplified using PCR and cloned into pET-28a(+) vector (Novagen) containing C-terminal His_6_-tag. Cloned plasmid were transformed into *Escherichia coli* Rosetta 2(DE3)pLysS strain. Recombinant proteins were overexpressed by adding 0.5 m*M* IPTG (isopropyl 1-thio-ß-D-galactopyranoside) at O.D._600nm_ 0.5 and cells were further incubated at 20°C overnight (16 h) using Luria Broth culture media. Harvested cells were lysed by cell sonicator (SONICS) in lysis buffer (20 m*M* Tris-HCl, pH 7.5, 500 m*M* NaCl, 35 m*M* imidazole, and 1 m*M* PMSF (phenylmethylsulfonyl fluoride)). After incubation of cell lysate at 60°C for 10 minutes, cell debris and denatured proteins were removed by centrifugation at 35,000 xg for 50 minutes. Supernatant was applied to HiTrap Chelating HP column (GE Healthcare) for affinity chromatography. Loaded sample was eluted with elution buffer (20 m*M* Tris-HCl, pH 7.5, 500 m*M* NaCl, and 500 m*M* imidazole) following equilibration with washing buffer (20 m*M* Tris-HCl, pH 7.5, 500 m*M* NaCl, and 50 m*M* imidazole). Eluted sample was further purified by size-exclusion chromatography with HiLoad 16/600 Superdex 200 prep grade column (GE Healthcare) equilibrated with 20 m*M* Tris-HCl, pH 7.5, and 200 m*M* NaCl. Fractions containing TPI proteins were further purified by anion exchange chromatography with HiTrap Q HP column (GE Healthcare) after 2 X dilution of the fractions with 20 m*M* Tris-HCl, pH 7.5, and 100 m*M* NaCl. Linear gradient of 0–500 m*M* NaCl in 20 m*M* Tris-HCl, pH 7.5, was applied for elution. For the final purification step, second size-exclusion chromatography was performed with HiLoad 16/600 Superdex 200 prep grade column (GE Healthcare) equilibrated with 20 m*M* Tris-HCl, pH 7.5, and 200 m*M* NaCl for TaTPI or 50 m*M* HEPES (4-(2-hydroxyethyl)-1-piperazineethanesulfonic acid), pH 7.5, for MjTPI. For crystallization, purified TaTPI proteins were concentrated to 30 mg ml^-1^.

### Crystallization and structure determination

Initial crystals of apo-TaTPI were grown at 22°C by sitting drop vapor diffusion method with crystallization solution containing 2 *M* NaCl and 10% (w/v) polyethylene glycol 6000. Apo-TaTPI crystals suitable for diffraction data collection were grown at 4°C using hanging-drop vapor diffusion method after mixing apo-TaTPI proteins with 2 times volume of crystallization solution containing 0.5 *M* NaCl, 10% (w/v) polyethylene glycol 6000, and 0.1 *M* Tris-HCl, pH 8.5. Crystals of TaTPI in complex with glycerol-3-phosphate (G3P) were obtained from crystallization solution with 0.6 *M* NaCl, 9% (w/v) polyethylene glycol 6000, and 0.1 *M* Tris-HCl, pH 8.5, after incubating purified apo-TaTPI proteins with 20 m*M* G3P for 4 h at 4°C. For diffraction data collection, crystals were frozen in liquid nitrogen following cryoprotection by soaking TaTPI crystals in crystallization solution supplemented with 20% glycerol. X-ray diffraction data for apo- and G3P-bound complex TaTPI crystals were collected at BL-7A synchrotron beamline at the Pohang Light Source, Korea, and at BL-1A synchrotron beamline at the Photon Factory, Japan, respectively. Collected data were processed using *HKL*2000 program suite [15]. The crystal structure of apo-TaTPI was solved by molecular replacement method using MOLREP program [16], with the crystal structure of *Pyrococcus woesei* TPI (PDB ID: 1HG3) as a phasing model [14]. The crystal structure of G3P-bound TaTPI was solved with the apo-TaTPI structure as a phasing model. The initial model building was carried out by *WinCoot* program [17] and further refined with *REFMAC*5 program [18]. All refinement steps were monitored using *R*_free_ value based on 5.0% of independently saved reflections. The final model was evaluated with *MolProbity* program [19]. Data collection and refinement statistics are summarized in Table 1.

**Table 1:** Statistics for data collection, phasing, and model refinement.

Data collection^a^	TaTPI	G3P-bound TaTPI
Space group	P2_1_2_1_2_1_	P2_1_2_1_21
Cell dimensions		
a, b, c (Å)	75.49, 113.96, 114.80	72.63, 84.08, 143.54
α, β, γ (°)	90, 90, 90	90, 90, 90
**Data set**		
X-ray wavelength (Å)	1.0000	1.1000
Resolution (Å)^b^	50.00–1.94 (1.97–1.94)	50.00–2.17 (2.21–2.17)
Total / unique reflections	521,936 / 73,921	294,576 / 47,245
Mean *I/sigma(I)*	32.4 (3.3)	20.9 (3.0)
Multiplicity	7.1 (5.6)	6.2 (5.6)
Completeness (%)	99.0 (83.5)	99.9 (99.9)
*R*_*merge*_ (%)^c^	9.1 (62.5)	13.4 (59.0)
**Refinement**		
Resolution (Å)	50.00–1.94	50.00–2.17
*R*_*work*_ ^d^ / *R*_*free*_ ^e^ (%)	18.0 / 21.5	18.3 / 22.5
No. of non-hydrogen atoms / mean B-factor (Å^2^)		
Protein	6,996 / 32.9	6,640 / 34.2
Water	561 / 39.8	401 / 34.4
Other atoms	28 / 40.1	44 / 22.9
Poor rotamers (%)^f^	0.1	0.3
Ramachandran plot analysis (%)		
Most favored regions	98.0	96.1
Additional allowed regions	2.0	3.4
Disallowed regions	0	0.5
R.m.s.d. from ideal geometry		
Bond lengths (Å)	0.019	0.015
Bond angles (°)	1.648	1.550

^a^Data collected at the synchrotron BL-7A at the Pohang Light Source and BL-1 at the Photon Factory.

^b^Numbers in parentheses indicate the highest resolution shell.

^c^ *R*_*merge*_ = Σ_h_ Σ_i_ |*I*(*h*)_i_−<*I*(*h*)>| / Σ_h_ Σ_i_ *I*(*h*)_i_, where *I*(*h*) is the observed intensity of reflection h, and < *I*(*h*) > is the average intensity obtained from multiple measurements.

^d^ *R*_*work*_ = Σ | |*F*_*o*_|—|*F*_*c*_| | / Σ |*F*_*o*_|, where |*F*_*o*_| is the observed structure factor amplitude and |*F*_*c*_| is the calculated structure factor amplitude.

^e^ *R*_*free*_ = R-factor based on 5.0% of the data excluded from refinement.

^f^Values obtained using MolProbity.

### Analytical ultracentrifugation (AUC)

Equilibrium sedimentation studies were performed using Beckman ProteomeLab XL-A analytical ultracentrifuge in 20 m*M* Tris-HCl buffer, pH 7.5, and 200 m*M* NaCl at 20°C. Sedimentation data were collected at 280 nm using a six-sector cell at rotor speeds of 20,000 and 26,000 rpms with three different protein concentrations: 12.6 u*M* (0.31 mg ml^-1^), 16.8 u*M* (0.41 mg ml^-1^), and 21.0 u*M* (0.52 mg ml^-1^). All measured data fit well to a homogeneous dimer model and representative results measured at 26,000 rpm using 16.8 u*M* protein concentration are presented. TaTPI concentrations were calculated using e_280nm_ = 11,920 M^-1^cm^-1^ and molecular weight of 24,671 daltons. Time required for the attainment of equilibrium was established by running at given rotor speed until scans were invariant for 4 hours: this was achieved at most by 36 hours in six-sector cells using 130 ul of samples. Partial specific volume of TaTPI protein and buffer density were calculated using Sednterp [20]. The calculated partial specific volume at 20°C was 0.7417 cm^3^ g^-1^ and the buffer density was 1.00704 g cm^-3^. For data analysis by mathematical modeling using non-linear least-squares curve fitting, following functions were used for homogeneous (Eq 1) and interactive (Eq 2) models.

$$C_{r}=C_{b}exp[A_{p}M_{p}(r^{2}-{r_{b}}^{2})]+\varepsilon$$ (Eq. 1)

$$C_{r}=C_{b}exp[A_{p}M_{p}(r^{2}-{r_{b}}^{2})]+{C_{b}}^{n}exp[Ink+nA_{p}M_{p}(r^{2}-{r_{b}}^{2})]+\varepsilon$$ (Eq. 2)

$$A_{p}=(1-v\rho )\omega^{2}÷2RT$$

where *C*_r_ is the total concentration at the radial position *r*, *C*_b_ is the concentration of protein at the cell bottom, *M*_p_ is the molecular weight of protein monomer, *ε* is a baseline error term, *v* and *ρ* are the partial specific volume and the solution density, respectively, and *ω* is the rotor angular velocity. The *In*k value is a natural log for equilibrium constants for reversible models (1x-nx, where n is 2 & 3) on an absorbance scale. Selection of the best model was made by examining numbers of weighted sum of square and root mean square error values. Further data manipulation and data analysis by mathematical modeling were performed using MLAB [21].

### Circular dichroism (CD)

Temperature of maximum heat capacity (or half-denaturation temperature, Td) of TaTPI was measured by CD spectroscopy. Experimental sample was prepared following purification and concentration of TaTPI proteins to 0.4 mg ml^-1^ containing 50 m*M* HEPES, pH 7.5. CD traces of TaTPI were obtained at 222 nm using the J-1500 CD spectrometer (JASCO) at a scanning rate of 1°C min^-1^. The denaturation curve of TaTPI was analyzed by Kaleidagraph (Synergy Software) based on John and Weeks’s protocol [22].

Effect of pH variation on the secondary structure of TaTPI was also monitored by CD spectroscopy. pH of protein solutions containing 20 m*M* Tris-HCl and 200 m*M* NaCl was adjusted to desired values between pH 1.0 and pH 7.0 with HCl for CD measurements. The baseline signal was measured with a buffer containing 20 m*M* Tris-HCl, pH 7.5, and 200 m*M* NaCl. CD spectra of pH-titrated TaTPI were recorded and averaged over two scans between 200 to 260 nm using J-1500 CD spectrometer (JASCO). The secondary structure contents of TaTPI were calculated by Multivariate SSE Program (JASCO).

### Analytical size-exclusion chromatography

Analytical size-exclusion chromatography of TaTPI was performed with Superdex 200 10/300 GL column (GE Healthcare) following equilibration with 20 m*M* Tris-HCl, pH 7.5, and 200 m*M* NaCl. The applied protein were at concentrations of 0.9 and 4.5 mg ml^-1^. Standard proteins from Gel Filtration Standard (BIO-RAD) were applied to the column for calibration. The standard protein mixture contained thyroglobulin (M.W. 670 kDa), γ-globulin (M.W. 158 kDa), ovalbumin (M.W. 44 kDa), myoglobin (M.W. 17 kDa), and vitamin B_12_ (M.W. 1.35 kDa).

### Differential scanning calorimetry (DSC)

Maximum temperature of heat capacity of MjTPI was measured with VP-DSC differential scanning microcalorimeter (Malvern). Experimental sample was prepared following purification and concentration of MjTPI proteins to 1.6 mg ml^-1^ (65 u*M*). Sample buffer containing 50 m*M* HEPES, pH 7.5, was loaded into the DSC cell after degassing in an evacuated chamber for 5 minutes and reference data were measured with scan rate of 0.5°C min^-1^. Experimental temperature was increased in the range from 50 to 120°C. After sample buffer scan reached equilibrium, MjTPI proteins were carefully loaded into the cell following degassing and experiments were performed under the same condition as for the reference. Heat capacity curve was plotted using the Origin software (Malvern).

### Accession numbers

The structure coordinates and structure factors for apo- and G3P-bound TaTPI have been deposited in PDB under the accession code 5CSR and 5CSS, respectively.

## Results and Discussion

### Overall structures of apo- and glycerol-3-phosphate-bound TaTPI

**Fig 1:**
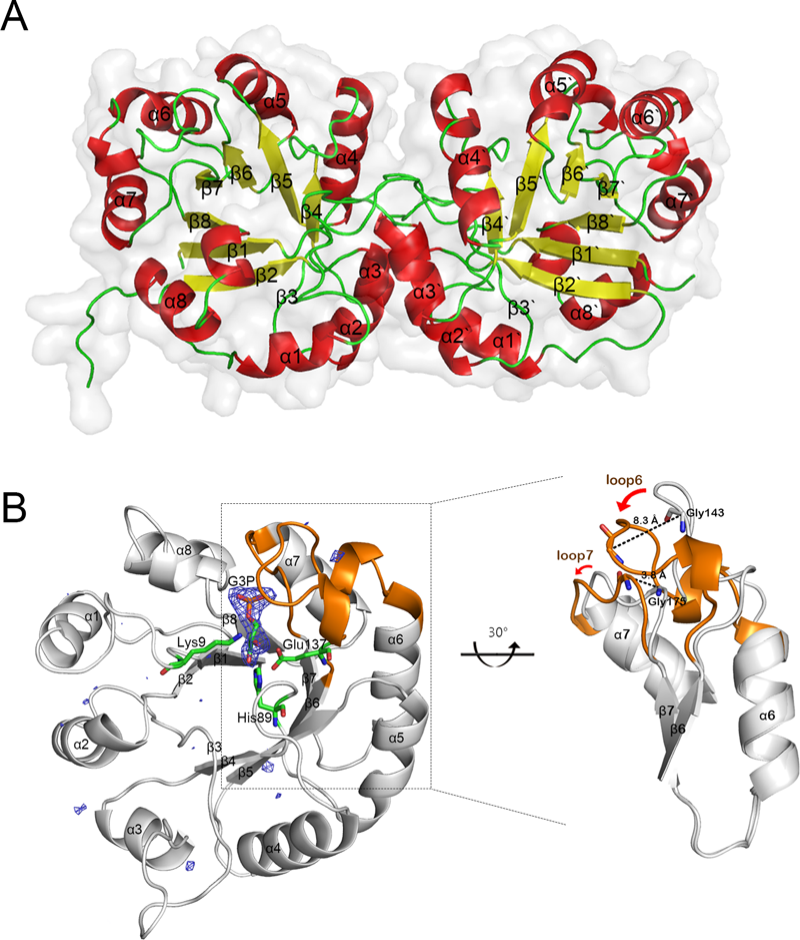
Overall structures of apo- and G3P-bound TaTPI. (A) Homodimer of apo-TaTPI is shown in carton representation, where α-helix, β-strand, and loop are colored in red, yellow, and green, respectively. (B) G3P-bound TaTPI. Only monomer of homodimeric G3P-bound TaTPI is demonstrated to emphasize a conformational change compared with apo-TaTPI. Extra positive electron density in *F*_*o*_ *-F*_*c*_ omit map contoured at 3.0 δ is shown as a blue mesh, which is modelled as G3P later. Amino acid residues interacting with G3P in catalytic site and G3P are shown as stick model; carbon, oxygen, phosphorus, and nitrogen atoms are colored in green, red, orange, and, blue, respectively. Loop 6 and loop 7 regions, which show a distinctive conformational change upon binding of G3P, are represented in orange and magnified for clarity.

The crystal structures of apo-TaTPI and its complex with glycerol-3-phosphate (G3P), an analogue of the substrate glyceraldehyde-3-phosphate, have been determined at 1.94 and 2.17 Å resolution, respectively. *R*_*work*_/*R*_*free*_ values for the final models of apo- and G3P-bound TaTPI were 18.0%/21.5% and 18.3%/22.5%, respectively (Table 1). Crystal structures of apo- and G3P-bound TaTPI contain four copies of TaTPI monomer in the asymmetric unit, comprising two homodimers. The overall structure of TaTPI protomer confirms to the canonical TIM-barrel fold with eight α-helices and eight parallel β-strands from 216 amino acid residues (Fig 1A). The crystal structure of G3P-bound TaTPI reveals that G3P is well positioned at the active site of TaTPI with functionally conserved residues Lys9, His89, and Glu137 (Fig 1B). Glu137 is clearly demonstrated as a catalytic base, with its position being within 3 Å from O1 and O2 of G3P. An oxyanion hole, which contributes to the stabilization of transition state, is formed by NZ nitrogen of Lys9 and NE2 nitrogen of His89 with O2 oxygen of G3P. The phosphate group of G3P is perfectly coordinated through hydrogen bonds with backbone nitrogen atoms of Gly143, Gly175, Ala196, and Ser197 residues, including adjacent water molecules (S1 Fig).

Overall structures of apo- and G3P-bound TaTPI were similar with r.m.s.d. of 0.69 Å for 216 C_α_ positions, except G3P-bound area. The conformational changes in the loop 6 of ligand bound-TPIs have been reported in previously determined structures of TPIs from many species, including *Trypanosoma brucei*, *Gallus gallus*, *Geobacillus stearothermophilus*, *Leishmania mexicana*, and *Vibrio marinus* [12, 23–27]. Likewise, loop 6 located between β-strand 6 and helix 6 and loop 7 located between β-strand 7 and helix 7 showed remarkable conformational changes when G3P binds to apo-TaTPI. When compared with apo-TaTPI structure, Gly143 located in loop 6 and Gly175 located in loop 7 moved toward G3P by 8.3 and 3.8 Å, respectively (Fig 1B). Consequently, residue Ala174 is pushed to a disallowed region of the Ramachandran plot in the G3P-bound form, an amino acid residue within loop 7 that is displaced by the ligand binding. G3P binding triggers the conformational change and induces the transformation of TaTPI from open to closed form, securing the catalytic site from bulk solvent to maintain efficient catalytic activity. In addition, water molecules around the active site of apo-TaTPI are expelled from the active site and would be replaced with substrate upon substrate binding.

### Unique dimeric conformation of TaTPI

**Fig 2:**
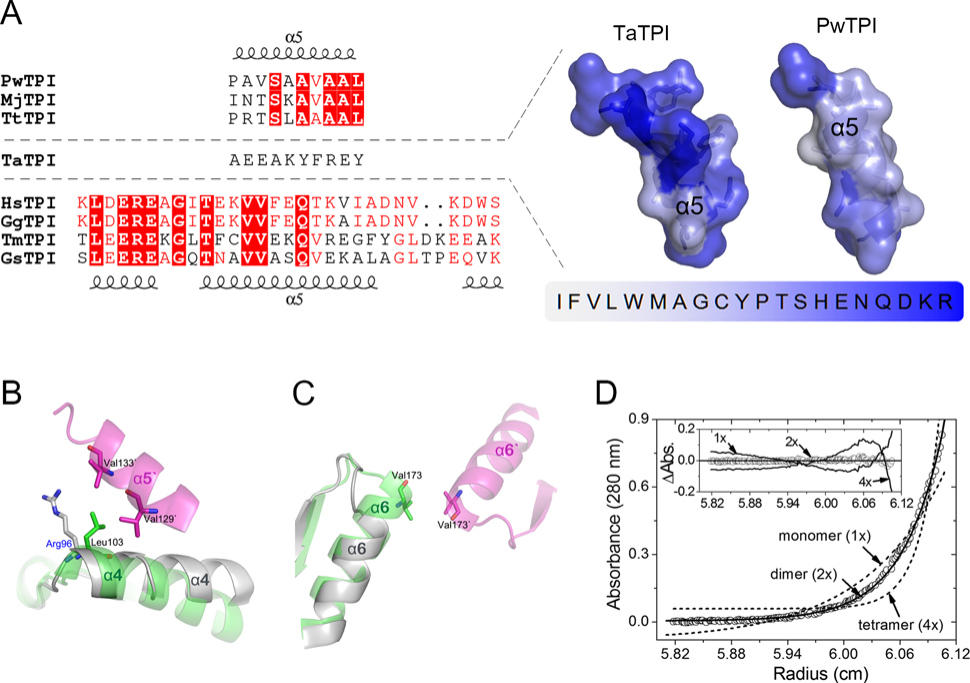
Dimeric conformation of TaTPI. (A) Structure-based sequence alignment of helix 5 region among TPIs from *Thermoplasma acidophilum* (TaTPI), *Pyrococcus woesei* (PwTPI), *Methanocaldococcus jannaschii* (MjTPI), *Thermoproteus tenax* (TtTPI), *Homo sapiens* (HsTPI), *Gallus gallus* (GgTPI), *Thermotoga maritima* (TmTPI), and *Geobacillus stearothermophilus* (GsTPI). Strictly conserved amino acid residues are highlighted in red shaded boxes and moderately conserved amino acid residues are colored in red. Helix 5 regions of TPIs from *Thermoplasma acidophilum* (PDB ID: 5CSR) and *Pyrococcus woesei* (PDB ID: 1HG3) are shown as cartoon representation with transparent surfaces, where amino acid residues are colored according to normalized consensus hydrophobicity scale [31]. (B) and (C) Structural comparison of helix 4 and helix 6 in TaTPI (gray) and PwTPI (green and magenta, each from different monomers). The important amino acid residues in tetrameric interface are shown as stick model. (D) Analytical ultracentrifugation experiment of TaTPI. Sedimentation equilibrium distribution (circle) of TaTPI at 26,000 rpm and 20°C is plotted as circle. Concentration of TaTPI was 16.8 u*M* (0.41 mg ml^-1^) in 20 m*M* Tris-HCl, pH 7.5, and 0.2 *M* NaCl. Solid line is a fitting line for a homogeneous dimer (2x) model and dotted lines are fitting lines for homogeneous monomer (1x) and tetramer (4x) models. Calculated molecular weight for TaTPI monomer from its amino acid compositions is 24,671 daltons. Inset graph shows distributions of the residuals for homogeneous 1x, 4x (solid lines), and 2x (circle) models, respectively. The random distribution of the residuals for the 2x model indicates that TaTPI exists as homogeneous dimers in solution.

*T*. *acidophilum* belongs to thermoacidophilic euryarchaeota and TaTPI also shares high amino acid sequence similarity to other thermostable TPIs from *P*. *woesei*, *T*. *tenax*, and *M*. *jannaschii*, all of which adopt tetrameric conformation that renders them stable at high temperatures [14, 28, 29]. Helix 4 and helix 5 of thermostable TPIs have been known to play important roles in the tetramer formation via hydrophobic interactions [14]. However, crystal structures of apo- and G3P-bound TaTPI form a dimer and reveal a remarkable difference in helix 5 of TaTPI from other thermostable TPIs. Helix 5 of TaTPI is mainly composed of charged-amino acid residues (AEEAKYFREY) instead of hydrophobic residues found in other thermostable tetrameric TPIs. Structure-based sequence alignment of TaTPI with other TPIs shows that TaTPI resembles bacterial dimeric TPIs rather than tetrameric TPIs from thermostable archaea (Fig 2A).

Structural differences between TaTPI and other tetrameric archaeal TPIs are observed not only in helix 5 but also in adjacent α-helices. In tetrameric archaeal TPIs, the N-terminus of helix 4 and the C-terminus of helix 6 play important roles in tetrameric interaction via hydrophobic effects and hydrogen bonds [14]. The N-terminus of helix 4 of tetrameric archaeal TPIs contains conserved Leu (Leu103 in PwTPI numbering) that contributes to hydrophobic effects and hydrogen bonds with the other side of helix 4 and helix 5 for its tetrameric assembly. In the case of TaTPI, Leu in the N-terminus of helix 4 of tetrameric archaeal TPIs is replaced with Arg96, which deprives the hydrophobic effects for tetramer formation (Fig 2B). Furthermore, TaTPI has shorter helix 6 than that of tetrameric archaeal TPIs, resulting in dissipated hydrophobic effects between two C-termini from each helix 6 of its accompanying dimeric partner, Val173 in PwTPI (Fig 2C). Consequently, changes in amino acid composition of helix 5, thus in electrostatic surface, and slight modification of secondary structures in helix 4 and helix 6 seem to play subtle roles in dimer formation.

In the crystal structure of apo-TaTPI, Cys50 in helix 2 of each homodimer drew our attention since it interacts with the other Cys50 from adjacent TaTPI homodimer via sulfur-containing hydrogen bonds, not disulfide bonds. Thus, we needed to re-confirm the oligomeric status of apo-TaTPI to make it sure whether the interaction of Cys-Cys is physiologically relevant or crystallographic artefact. To verify the oligomeric state of apo-TaTPI in solution, analytic ultracentrifugation analysis (AUC) was performed. Oligomeric state of apo-TaTPI in solution was investigated by equilibrium sedimentation technique at two speeds and three concentrations. Fig 2D shows the data and fits analyzed by using Eq. 1 for homogeneous 1x, 2x, and 4x models at ultracentrifugal speed of 26,000 rpm. The weighted root-mean-square errors (RMS) for the 1x and 4x fits were 5.01 x 10^−2^ and 6.90 x 10^−2^, respectively. In contrast to these models, dimer (2x) model gave much improved RMS value of 7.93 x 10^−3^. Residual distribution plot (Fig 2D inset) also supports that the apo-TaTPI forms dimer in solution. Analysis at 20,000 rpm (data not shown) also gave a better RMS value of 5.76 x 10^−3^ for 2x model than those for 1x (3.90 x 10^−2^) and 4x (5.42 x 10^−2^) models. Mixture or reversible models were also investigated but there was no indication of the possibility. Data analysis using the reversible model (Eq 2) gave large negative *In*k values for monomer-dimer (1x-2x) and monomer-trimer (1x-3x) models and much higher RMS values (~10^−2^), so the reversible models for monomer-dimer (1x-2x) and monomer-trimer (1x-3x) equilibrium would not be the case for TaTPI. These results strongly indicate that apo-TaTPI exits as homogeneous dimer in solution. In addition, analytical size-exclusion chromatography results of apo-TaTPI, which were confirmed at two different TaTPI protein concentrations (0.9 and 4.5 mg ml^-1^), also supported the dimeric conformation in solution (S2 Fig).

### Structural stability of TaTPI under extreme condition

*T*. *acidophilum* thrives in harsh environments such as high temperature and extremely acidic condition. TaTPI is also expected to function correctly at high temperature or in very low pH condition when physiological barriers are affected by various stresses. To elucidate the structural stability of TaTPI in extremely acidic condition and at high temperature, we carried out circular dichroism (CD) spectroscopy experiments.

As for the structural stability of TaTPI in extremely acidic condition, secondary structure changes of pH-titrated TaTPI were monitored using CD spectroscopy. Normally, intracellular environment is well kept from extracellular stresses such as abrupt pH change. So, most of intracellular proteins experience normal physiological conditions and function accordingly. TaTPI maintained its folded structure under extremely acidic condition (pH 1–2) as in neutral pH range (S3 Fig). In the case of TaTPI, secondary structure contents under extremely acidic condition seem to change slightly with sustained folded structure. The content of α-helix in the TaTPI tends to increase with decreasing pH, whereas the content of β-strand decreases. These results suggest that TaTPI is designed to function normally even in cases of unexpected pH drop.

The temperature of maximum heat capacity (or half-denaturation temperature, Td) of TaTPI was measured by CD spectroscopy. The denaturation curve of TaTPI was analyzed by Kaleidagraph (Synergy Software) based on John and Weeks’s protocol [22]. The Td value for TaTPI is 74.6°C (S4 Fig), which is comparable with that of TPI from *Geobacillus stearothermophilus* (GsTPI), a biological indicator for the validation of sterilization processes, (T_d_: 76°C) [30]. In the case of GsTPI, a large number of prolines (5.2%), replacement of asparagine by histidine within the active site to prevent deamidation, the smallest cavity number and volume, and a large buried hydrophobic surface have been shown to contribute for the thermostability [12]. *Thermotoga maritima*, a hyperthermophilic bacterium, has been known to have TPI with the highest T_d_ value of 102°C [30], resulting from a large number of salt bridges and extensive hydrophobic patches from tetramer conformation [8]. Using DSC, we found the Td app value of MjTPI to be 107.1°C and it is the highest Td value among reported TPIs so far (Table 2; S5 Fig). When compared with TPIs mentioned above, TaTPI has smaller number of prolines (4.2%), larger cavity volume, and less buried hydrophobic surface than GsTPI, nor does it adopt tetrameric conformation.

**Table 2:** Half-denaturation temperatures of TPIs.

	The half-denaturation temperature (T_d,_°C)	Reference
TaTPI	74°C	This manuscript
MjTPI	107°C	This manuscript
TmTPI	102°C	Alvarez et al., 1999 [30]
GsTPI	76°C	Alvarez et al., 1999 [30]
PfTPI	65°C	Gopal et al., 1999 [33]
HsTPI	55°C	Mainfroid et al., 1996 [34]
TbTPI	44°C	Borchert et al., 1993 [35]

Ta, *Thermoplasma acidophilum*; Mj, *Methanocaldococcus jannaschii*; Tm, *Thermotoga maritima*; Gs, *Geobacillus stearothermophilus*; Pf, *Plasmodium falciparum*; Hs, *Homo sapience*; Tb, *Trypanosoma brucei*.

### Proposal of TPI stabilization patches

**Fig 3:**
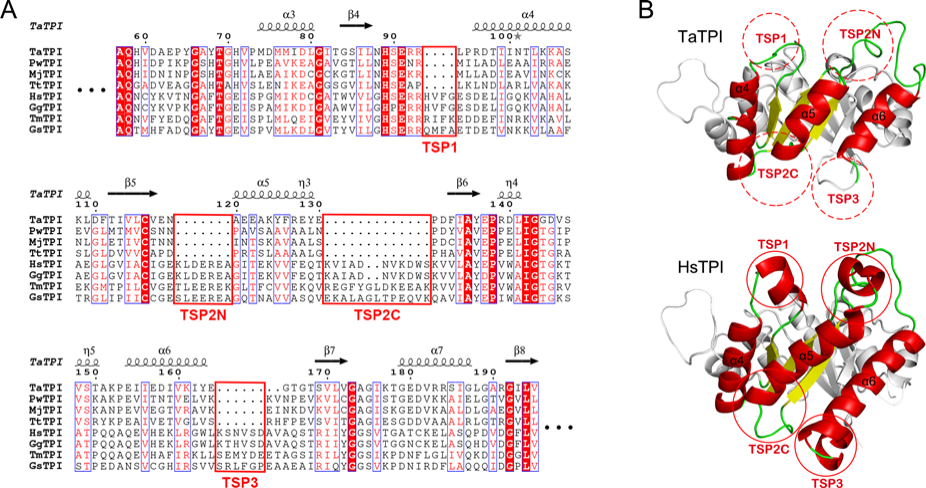
TPI stabilization patches (TSPs). (A) Structure-based sequence alignment of TaTPI with other TPIs from *Pyrococcus woesei* (PwTPI), *Methanocaldococcus jannaschii* (MjTPI), *Thermoproteus tenax* (TtTPI), *Homo sapiens* (HsTPI), *Gallus gallus* (GgTPI), *Thermotoga maritima* (TmTPI), and *Geobacillus stearothermophilus* (GsTPI). Strictly conserved amino acid residues are highlighted in red shaded boxes and moderately conserved amino acid residues are colored in red. Conserved residues are enclosed in blue boxes and TSP regions are enclosed in red boxes. The alignment figure was prepared using *ESPript* program [32]. (B) Structural comparison of TaTPI and HsTPI. The red dotted and solid circles represent TSP regions in TaTPI and HsTPI, respectively.

TaTPI is composed of 216 amino acid residues and approximately 10% shorter in length than other TPIs from bacterial and eukaryotic species, which is a common feature in archaeal TPIs. According to the results of our structure-based sequence alignment for TPIs, helix 4, helix 5, and helix 6 are regions that mainly account for variation in amino acid composition, structural stability, and the oligomeric status of TPIs. To systematically validate the stabilization factors of TaTPI, we name regions of distinctive differences as TPI stabilization patches (TSPs) (Fig 3A). TSP1 is helix 4 region and TaTPI lacks a short helical N-terminus in this patch. Consequently, TaTPI forms a more compact structure. TSP2 exhibits a major structural discrepancy in that 18 amino acid residues are missing in helix 5 region of TaTPI, compared with other dimeric TPIs. In TSP2 area, truncated helix 5 (TSP2C) and lack of a short helix in its N-terminus (TSP2N) also make TaTPI tighter in their overall structure. Lastly, TSP3 arises from helix 6 region and helix 6 is trimmed along with a short α-helix in its C-terminus (Fig 3B). All the TSPs of TaTPI contribute to the formation of more compact dimeric structure.

The TSPs are not exclusive of TaTPI. Other tetrameric archaeal TPIs also have the TSPs. We speculated that other tetrameric archaeal TPIs could be more thermostable than TaTPI since they adopt tetrameric conformation and contains TSPs. To validate our speculation, we cloned and purified *M*. *jannaschii* TPI (MjTPI), a tetrameric archaeal TPI, and measured the Td app value of MjTPI. The Td app value of MjTPI was 107.1°C, which is the highest Td app value among reported TPIs so far. The result suggest that both higher oligomerization status and the TSPs could be important factors for thermostability of TPIs. TaTPI would keep its structural and functional integrity at high temperature through a compact dimeric conformation from contributing TSPs. Crystal structures of apo- and G3P-bound TaTPI and defined stability determining factors of TPIs would provide clear insights on engineering more stable TIM-barrel fold proteins which make up a large protein family and play pivotal role in metabolic pathways.

## Supporting Information

S1 Fig Binding modes of G3P with key amino acid residues of TaTPI. LIGPLOT diagram is used for representation of active site in the G3P-bound TaTPI. Carbon, nitrogen, oxygen, and phosphorus atoms are shown in black, blue, red, and magenta, respectively. Hydrogen bonds and oxyanion hole between G3P and TaTPI are shown as green and red dotted line, respectively.
(TIF)

S2 Fig Analytic size-exclusion chromatography of TaTPI. TaTPI protein samples at two different concentrations (A: 4.5 mg ml^-1^, B: 0.9 mg ml^-1^) were applied to Superdex200 10/300 GL column. Chromatograms of TaTPI and gel filtration standard were shown as blue and red lines, respectively.
(TIF)

S3 Fig Circular dichroism spectra for TaTPI at variable pH conditions. (A) CD spectra of pH-titrated TaTPI (pH 1.0–7.0) were measured from 200 to 260 nm. (B) The secondary structure contents of pH-titrated TaTPI were calculated from Multivariate SSE Program (JASCO).
(TIF)

S4 Fig Thermal denaturation curve of TaTPI. The black line represents baseline-subtracted and normalized raw data. The red line indicates the best fits of the raw data. The maximum temperature of heat capacity (*T*_*d*_) was calculated according to the best fits of the raw data.
(TIF)

S5 Fig DSC thermogram of MjTPI. The black circle represents baseline-subtracted and normalized raw data. The black line indicates the best fits of the raw data. The maximum temperature of heat capacity (*T*_*d*_) was calculated based on the best fits of the raw data.
(TIF)

## Abbreviations

TaTPI: triosephosphate isomerase from *Thermoplasma acidophilum*
PDB: Protein Data Bank
G3P: glycerol-3-phosphate
