# Supplementary figures and images for: Structure and Stability of the Dimeric Triosephosphate Isomerase from the Thermophilic Archaeon Thermoplasma acidophilum
Source: PLoS One. 2015 Dec 28;10(12):e0145331. doi: 10.1371/journal.pone.0145331 (PMC4692482; doi:10.1371/journal.pone.0145331)

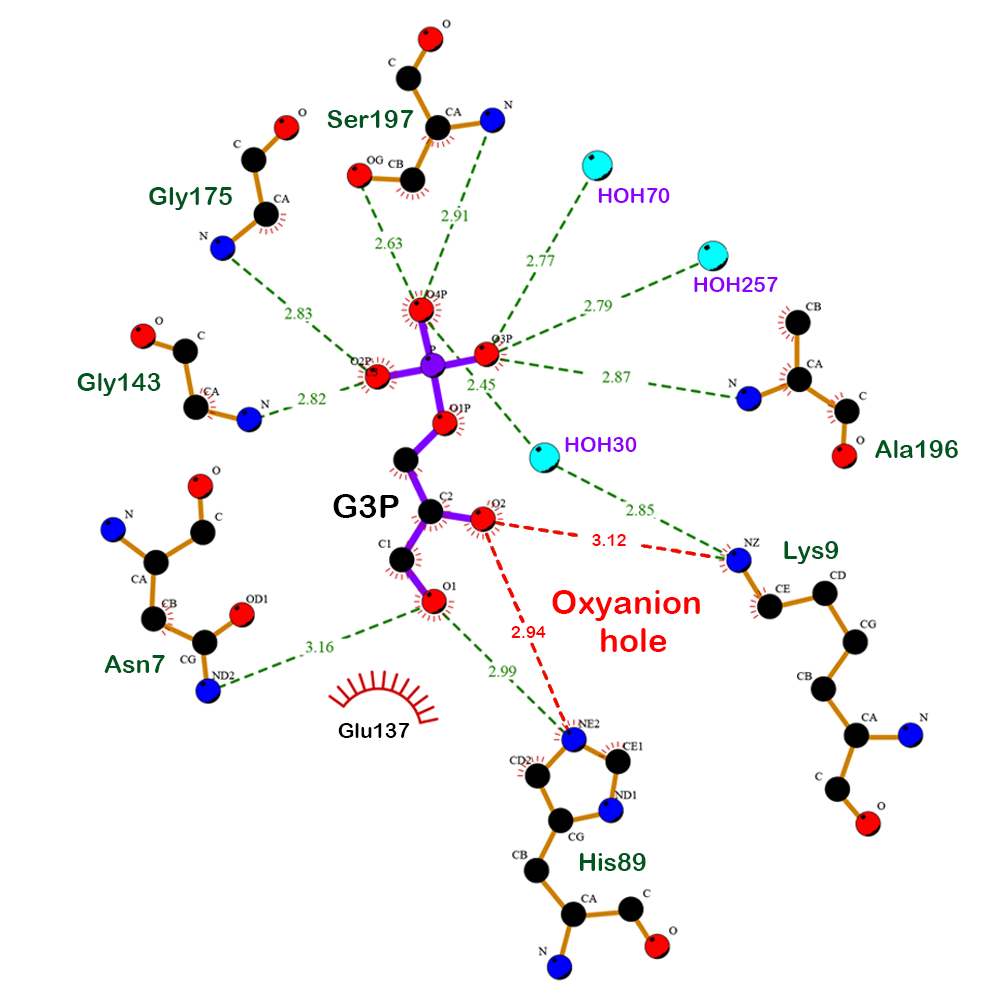

Supplement: S1 Fig — LIGPLOT diagram is used for representation of active site in the G3P-bound TaTPI. Carbon, nitrogen, oxygen, and phosphorus atoms are shown in black, blue, red, and magenta, respectively. Hydrogen bonds and oxyanion hole between G3P and TaTPI are shown as green and red dotted line, respectively. (TIF) [file pone.0145331.s001.tif]

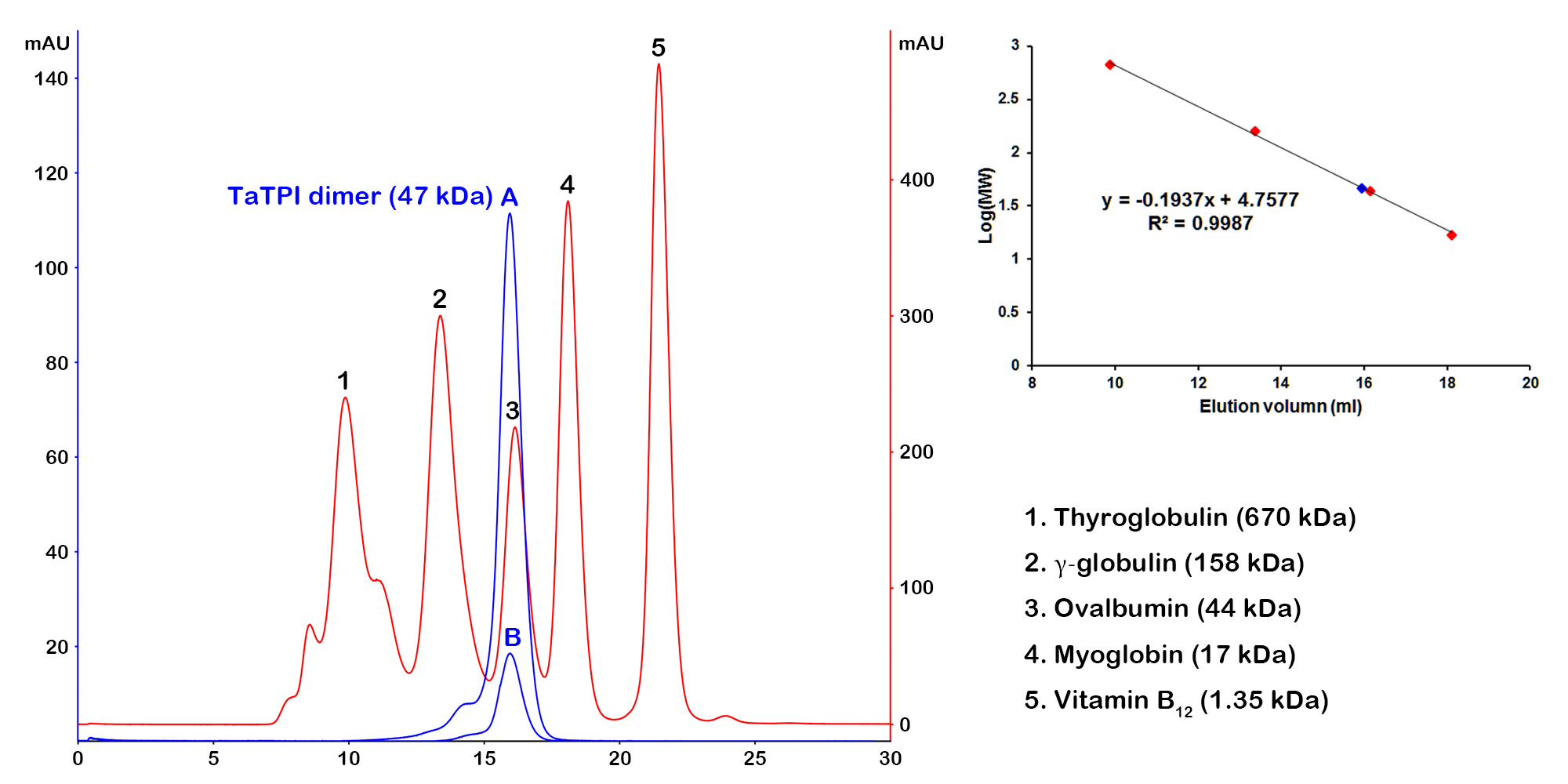

Supplement: S2 Fig — TaTPI protein samples at two different concentrations (A: 4.5 mg ml-1, B: 0.9 mg ml-1) were applied to Superdex200 10/300 GL column. Chromatograms of TaTPI and gel filtration standard were shown as blue and red lines, respectively. (TIF) [file pone.0145331.s002.tif]

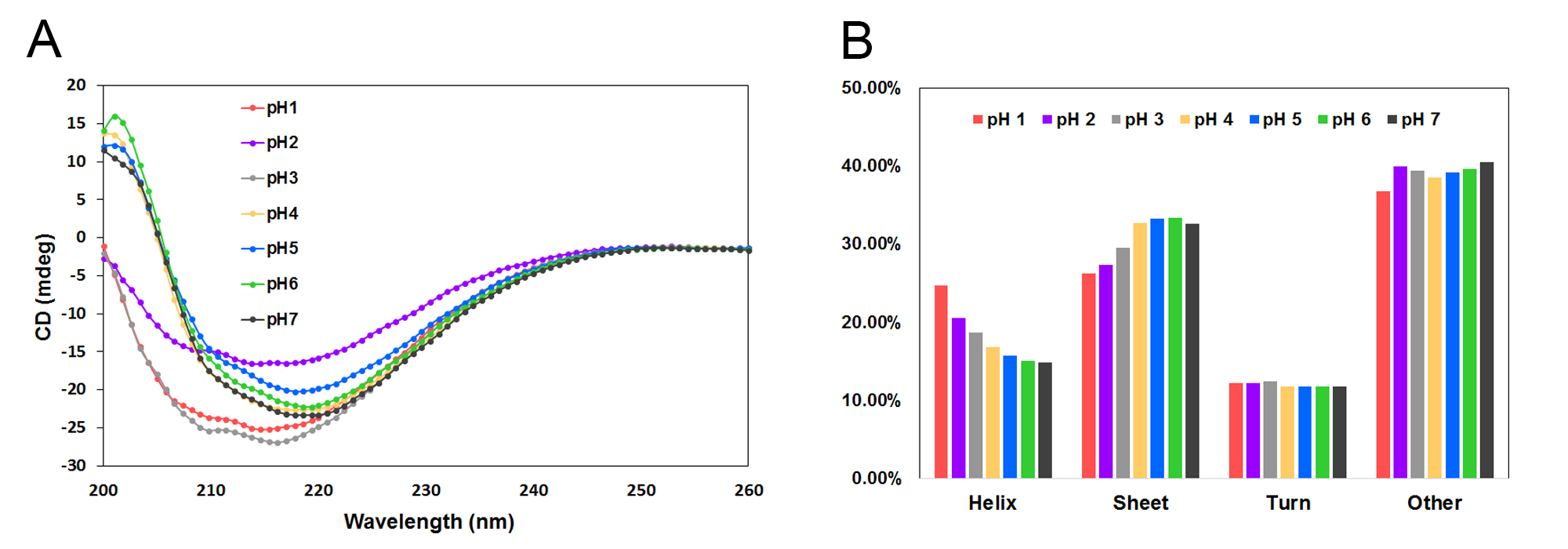

Supplement: S3 Fig — (A) CD spectra of pH-titrated TaTPI (pH 1.0–7.0) were measured from 200 to 260 nm. (B) The secondary structure contents of pH-titrated TaTPI were calculated from Multivariate SSE Program (JASCO). (TIF) [file pone.0145331.s003.tif]

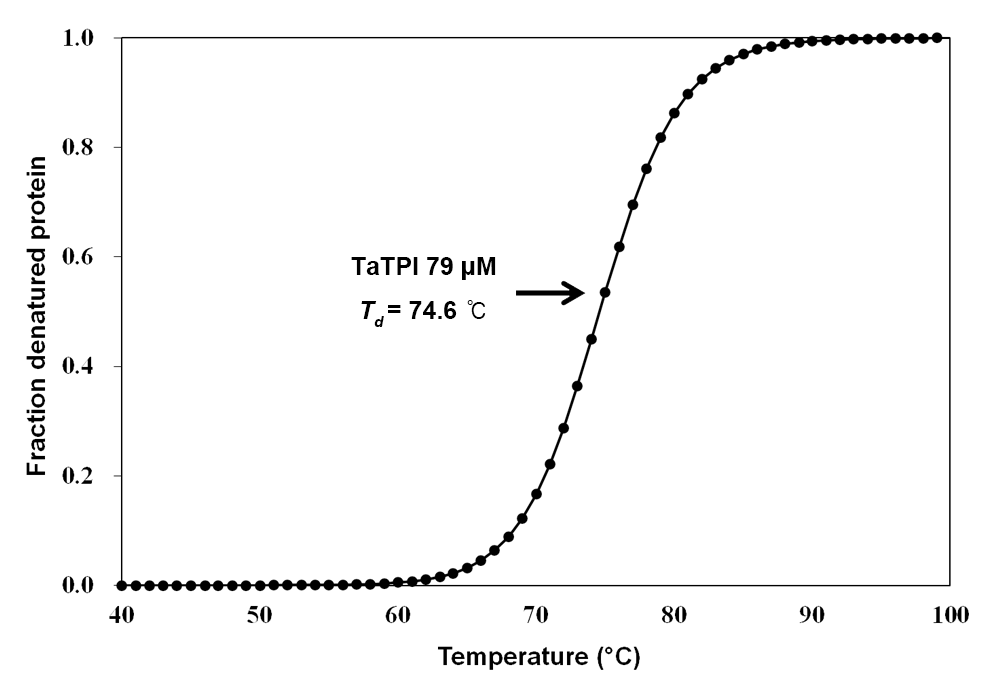

Supplement: S4 Fig — The black line represents baseline-subtracted and normalized raw data. The red line indicates the best fits of the raw data. The maximum temperature of heat capacity (T d) was calculated according to the best fits of the raw data. (TIF) [file pone.0145331.s004.tif]

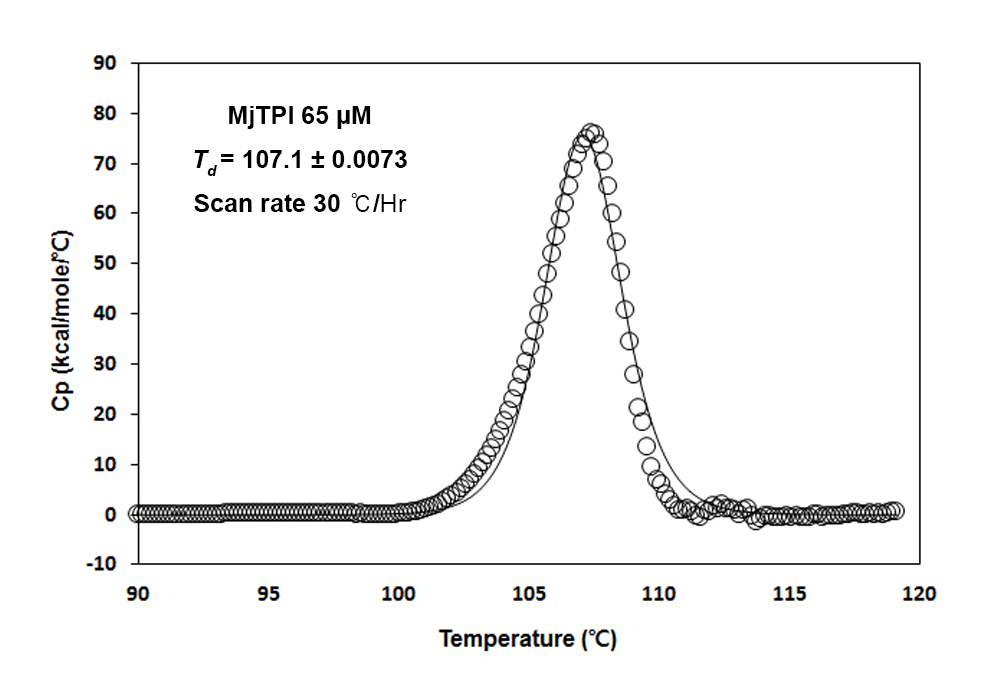

Supplement: S5 Fig — The black circle represents baseline-subtracted and normalized raw data. The black line indicates the best fits of the raw data. The maximum temperature of heat capacity (T d) was calculated based on the best fits of the raw data. (TIF) [file pone.0145331.s005.tif]
